# Learning the pattern of epistasis linking genotype and phenotype in a protein

**DOI:** 10.1038/s41467-019-12130-8

**Published:** 2019-09-16

**Authors:** Frank J. Poelwijk, Michael Socolich, Rama Ranganathan

**Affiliations:** 1 cBio Center, Department of Data Sciences, Dana-Farber Cancer Institute, 360 Longwood Avenue, Boston, MA 02215 USA; 2 Center for Physics of Evolving Systems, Department of Biochemistry & Molecular Biology, The Pritzker School for Molecular Engineering, University of Chicago, 929 East 57th Street, Chicago, IL 60637 USA

**Keywords:** Computational biology and bioinformatics, Molecular evolution, Epistasis, Epistasis, Synthetic biology

## Abstract

Understanding the pattern of epistasis—the non-independence of mutations—is critical for relating genotype and phenotype. However, the combinatorial complexity of potential epistatic interactions has severely limited the analysis of this problem. Using new mutational approaches, we report a comprehensive experimental study of all 2^13^ mutants that link two phenotypically distinct variants of the *Entacmaea quadricolor* fluorescent protein—an opportunity to examine epistasis up to the 13^th^ order. The data show the existence of many high-order epistatic interactions between mutations, but also reveal extraordinary sparsity, enabling novel experimental and computational strategies for learning the relevant epistasis. We demonstrate that such information, in turn, can be used to accurately predict phenotypes in practical situations where the number of measurements is limited. Finally, we show how the observed epistasis shapes the solution space of single-mutation trajectories between the parental fluorescent proteins, informative about the protein’s evolutionary potential. This work provides conceptual and experimental strategies to profoundly characterize epistasis in a protein, relevant to both natural and laboratory evolution.

## Abstract (web summary)

Epistasis underlies the complexity of genotype-phenotype maps. Here, the authors analyze 8,192 mutants that link two phenotypically distinct variants of the *Entacmaea quadricolor* fluorescent protein, and show the existence, but also the sparsity, of high-order epistatic interactions.

## Introduction

The central properties of proteins—folding, biochemical function, and evolvability—arise from a global pattern of cooperative energetic interactions between amino acid residues. When introducing amino acid substitutions in a protein, cooperativity manifests itself as context-dependence of the effects of those mutations, or epistasis^1^. Knowledge of the extent and distribution of epistasis in a protein is essential for understanding its evolution. For example, when a certain functional improvement requires a combination of mutations that are individually unfavorable, no single-mutation trajectory exists that increases fitness at each step, and evolution towards the new functionality will be hampered^2–4^. Being able to uncover epistasis is relevant for the reconstruction of phylogenetic trees^5^ and for estimating the evolutionary potential of antibiotic resistance genes^6,7^ and viruses^8^, but also for protein engineering efforts that make use of directed evolution: information on epistatic architectures should prove useful in the selection of evolvable templates^9,10^, in focusing mutations to highly-epistatic regions of a protein^11^, or in identifying cooperative units for DNA shuffling experiments^12,13^.

However, the challenge of mapping epistasis is extraordinarily complex. Epistasis can occur due to nonlinearities in any process linking genotype and fitness and can occur at the pairwise level (two-way) or extend to a series of higher-order terms (three-way, four-way, etc.) that describes the full extent of possible interactions^14–17^. As a consequence, the number of potential epistatic interactions grows exponentially with the number of positions in a protein, a combinatorial problem that becomes rapidly inaccessible to any scale of experimentation. Indeed, the theoretical complexity of this problem is such that it is not feasible or rational to propose an exhaustive mapping of epistasis for any protein.

**Fig. 1:**
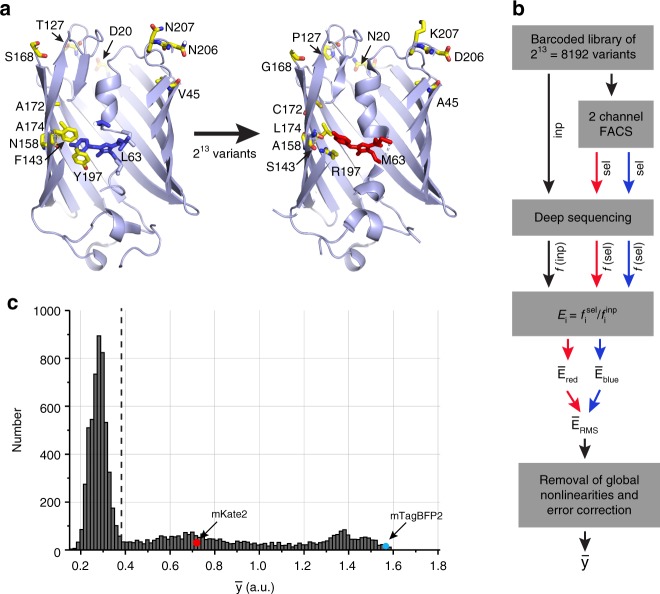
Combinatorial mutagenesis and data collection. **a** mTagBFP2 (left) and mKate2 (right) are blue and red variants, respectively, of the *Entacmaea quadricolor* fluorescent protein eqFP611^18^ that differ by 13 amino acid substitutions (12 shown; the 13th is at position 231, at the C-terminal end of the molecule and is not shown in the crystal structure). This defines a total sequence space linking the two of 2^13^ = 8192 variants. **b** A schematic of the experimental protocol in which every variant is assigned a quantitative phenotype ($\bar y$)—brightness, a combination of fluorescence in both red and blue channels (see “Methods” for details). The phenotype is computed such that the independent action of mutations outside of the selection threshold corresponds to additivity. **c** The distribution of phenotypes for all 8192 variants; the dashed line corresponds to the detection threshold for fluorescence

How, then, can we practically characterize the architecture of epistatic interactions between amino acids? We reasoned that a strategy may emerge from a focused experimental case study in which we make all possible combinations of mutations within a limited set of positions within a protein—a dataset from which we can directly determine the extent of epistasis and explore possible simplifying methods. As a model system, we chose the *Entacmaea quadricolor* fluorescent protein eqFP611^18^, a protein in which spectral properties and brightness represent easily measured, quantitative phenotypes with a broad dynamic range. Recently, two variants of eqFP611 have been reported, one bright deep-red (mKate2, *λ*_ex_ = 590 nm, *λ*_em_ = 635 nm^19^) and one bright blue (mTagBFP2, *λ*_ex_ = 405 nm, *λ*_em_ = 460 nm^20^), that are separated by thirteen mutations (Fig. 1a); we will refer to these as the “parental” genotypes.

By developing new technologies for high-throughput combinatorial mutagenesis and quantitative phenotyping, we explored the full space of 2^13 ^= 8192 variants comprising the parental genotypes and all possible intermediates between them. These data reveal a broad range of high-order epistatic interactions between mutations, suggesting great complexity in the relationship between genotype and phenotype. However, we find that epistasis is also highly sparse compared to theoretical limits, a property that opens up the use of powerful computational techniques for uncovering the epistatic architecture with practical experimental or sequence-based approaches. If sparsity is general, this observation suggests an approach for accurate phenotypic predictions of unobserved mutants based on phenotypic measurements of a limited set of genotypes, which we illustrate using our model system. Finally, we obtain a view of the model protein’s evolutionary potential: we find that the observed high-order epistasis greatly limits the number of viable single-mutation trajectories between the blue and red variants of eqFP611, but not so much as to preclude paths in which fluorescence is maintained throughout. The most severe epistasis occurs at mutational steps involved in the actual color switch, indicating a strong cooperativity between the fluorophore and its immediate environment.

## Results

### A complete combinatorial mapping of phenotypes

We developed an efficient iterative gene synthesis approach to simultaneously construct and barcode the full library of 8192 fluorescent protein (FP) variants that represents the parental genotypes mKate2^19^ and mTagBFP2^20^ and all possible intermediates (Fig. 1b, Supplementary Fig. 1, and “Methods”). This strategy makes it possible to readout the identity of every combination of mutations simply by high-throughput DNA sequencing of the barcode region—a method that should be generally valuable for studies of high-order epistasis. We expressed the library in *Escherichia coli*, carried out two-color fluorescence activated cell sorting (FACS) to select variants with brightness above a threshold, deep sequenced the input and selected libraries, and computed the frequency *f*_*a*_ of every FP allele *a* in the sorted population relative to the input population—an enrichment score (Fig. 1b). The color channels are normalized by the measured spectral properties of the parental red and blue proteins and are combined to produce a single quantitative phenotype–*y*—that reports fluorescence brightness (Fig. 1b, c, “Methods”, and Supplementary Fig. 2). This measure integrates various underlying biophysical properties—extinction coefficient, quantum yield, protein expression—and is therefore a rich phenotype for characterizing epistatic effects of mutations, even though other properties such as maturation half-time and some detailed spectral properties are not explicitly considered here.

Global non-linearities are expected due to the experimental process and were minimized in a mechanistically unbiased manner using the procedure of linear-nonlinear optimization^21–23^, permitting the assessment of relevant epistatic interactions between mutations (see “Methods” and Supplementary Fig. 6 for robustness of the main conclusions to this process). The bottom line is a quantitative assignment of phenotypes for all 8192 variants in a form in which independence of mutational effects for reasons other than the internal cooperativities of amino acids and the single threshold selection for brightness corresponds to additivity in *y* (see Supplementary Fig. 13 for additional discussion on detection limits). The fact that the parental genotypes are brightly fluorescent but many of the intermediates are not (Fig. 1c) is a first indication that we can expect substantial epistasis between mutations linking the two.

### From phenotypes to epistasis

**Fig. 2:**
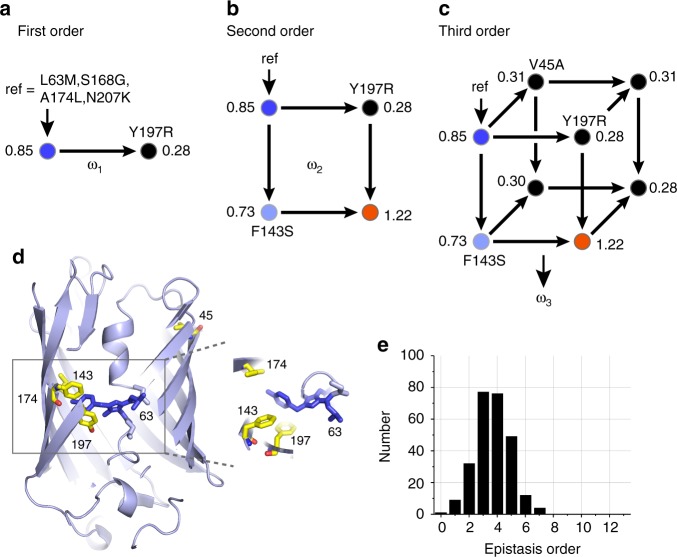
High-order epistasis in the sequence variations linking mTagBFP2 and mKate2. For illustration, panels **a**–**c** show the single reference form of epistasis taking an arbitrary genotype as the background (ref = L63M/S168G/A174L/N207K); circles indicate fluorescence color. All other panels indicate background-averaged epistasis. **a** First-order epistasis is simply the effect of a single mutation. For example, in the reference background, Y197R shows *ω*_1_ = 0.28–0.85 = −0.57, indicating loss-of-function. **b** Second order epistasis is the dependence of a first order term on a second mutation. Here, Y197R has a completely different effect in the background of F143S, and thus these two mutations display a large second order term (*ω*_2_ = 1.06). **c** Third-order epistasis is the dependence of a second order term on a third mutation. Here, the pairwise epistasis of Y197R and F143S is quenched in the background of V45A, indicating a large third-order term (*ω*_3_ = −1.07). **d** Positions involved in large epistatic interactions are shown, indicating sites both proximal and distal to the chromophore. **e** The distribution of the 260 statistically significant background-averaged epistatic terms (threshold, *P* < 0.01), showing a broad range of high-order interactions between amino acids, including terms up to the seventh order

From the full dataset of phenotypes, we computed the complete hierarchy—1-way, 2-way, 3-way, 4-way, etc.—of epistatic interactions between the thirteen mutated positions. Mathematically, epistasis is a transform (**Ω**) in which phenotypes ($\bar y$) of individual variants are represented as context-dependent effects of the underlying mutations ($\bar \omega$, Fig. 2a)^14,15^:

1 $$\bar \omega = {\mathbf{\Omega }}\bar y$$

For *N* positions with a single substitution at each position, $\bar y$ is a vector of 2^*N*^ phenotypic measurements in binary order (here, 2^13^) and $\bar \omega$ is a vector of 2^*N*^ corresponding epistatic interactions. A first-order epistatic term (*ω*_1_) is the phenotypic effect of a single mutation, a second order epistatic term (*ω*_2_) is the degree to which a single mutation effect is different in the background of second mutation, and a third-order epistasis (*ω*_3_) is the degree to which the second order epistasis is different in the background of a third mutation. Higher-order terms follow the same principle, such that an *n*th order epistatic term is the degree to which an *n*–1 order term depends on the context of yet another mutation, comprising a hierarchy of possible couplings between mutations. A key point is that $\bar \omega$ and $\bar y$ contain exactly the same information, but simply differ in its organization; $\bar y$ represents the phenotypes of individual variants while $\bar \omega$ represents the non-additive interactions between the mutations.

A few examples help to explain the concept of epistasis. If we take the variant L63M/S168G/A174L/N207K as an arbitrary reference state (*y*_ref_ = 0.85, blue fluorescence) the data show that introducing the mutation Y197R results in reduced brightness (*y* = 0.28) (Fig. 2a). The difference in these two values defines a first-order epistasis (*ω*_1_ = *y*_Y197R_–*y*_ref_ = −0.57). However, in the background of F143S, the effect of Y197R is entirely different; it shows increased brightness (*ω*_1|F143S_ =+ 0.49), with conversion to red fluorescence. This indicates a large second order epistatic term (*ω*_2_ = *ω*_1|F143S_–*ω*_1_ = 1.06, Fig. 2b), meaning that the effect of Y197R is context-dependent on F143S. This second order term is itself dependent on other mutations. For example, in the background of V45A, the second order epistasis between Y197R and F143S nearly vanishes (*ω*_2|V45A_ = −0.01), indicating a large third-order epistasis (*ω*_3_ = *ω*_2|V45A_–*ω*_2_ = −1.07, Fig. 2c). These findings show that Y197R, F143S, and V45A work as a cooperative unit whose contribution to phenotype cannot be broken down into a simple, additive contribution of the underlying mutational effects. Instead, prediction of phenotypes involving these mutations requires knowledge of their individual effects and epistatic interactions at all orders.

In the examples discussed above, the effects of mutations are computed relative to a single reference genotype—the background in which the mutations are made. But, why should we restrict the definition of epistasis in the local mutational neighborhood of an arbitrarily chosen reference sequence? A more general analysis would be to compute each epistatic term as an average over all possible genetic backgrounds. For example, the effect of Y197R (the first-order epistasis *ω*_1_) can be computed not just with respect to a single reference (Fig. 2c), but as the average of its phenotypic effect in the background of every one of the other 2^*N*–1^ genotypes. Similarly, one can define background averaged pairwise, three-way, and higher-order epistatic terms in which each term is averaged over all remaining genotypes. This view of epistasis is a global one, indicating the contribution of amino acids and interactions to protein function averaged over the full sequence space of variants^14,15,24–26^. We show the profound distinction of single-reference and background averaged epistasis below.

### The distribution of epistasis

We computed the background-averaged epistasis for the dataset of brightness phenotypes. Analysis of error propagation provides a rigorous basis for establishing the statistical significance of epistatic terms as a function of order (Supplementary Fig. 5 and “Methods”). At a significance threshold of *P* < 0.01, we identify 260 background averaged epistatic terms. These 260 terms are distributed as a function of order in a unimodal shape with a peak at 3rd–4th order, and include interactions up to the 7th order within the set of 13 mutated positions (Fig. 2e). The distribution is similar for both background averaged and single-reference forms of epistasis, and independent trials of the experiment demonstrates the robustness of determining the significant epistatic terms (Supplementary Fig. 7). Structurally, the epistatic terms involve not just residues in the local environment of the chromophore, but also includes positions (e.g., 45) located at a considerable distance at the opposite edge of the β-barrel (Fig. 2d). Indeed, the V45A mutation has the remarkable property of displaying a small effect on its own (*ω*_1_ = −0.08), but having much larger epistatic effects, for example, in modulating the pairwise coupling between Y197R and F143S (*ω*_3_ = −0.29).

In summary, the data indicate a broad range of high-order epistatic interactions between amino acid mutations that link the blue and red variants of the eqFP611 protein.

### Sparsity and prediction of phenotypes

At first glance, the finding of significant epistatic terms up to the seventh order would seem to pose an insurmountable practical challenge to the goal of relating genotype to phenotype in proteins. No studies are likely to make such measurements in general, and the scale of experimentation grows exponentially with protein size. However, the data also suggest the possibility of great sparsity in the number of significant epistatic terms, a finding that if confirmed, can open up practical approaches. For example, the 260 significant epistatic terms identified here represent only a small fraction of the 8192 possible terms. How much information is encoded in just these terms? To study this, we used the inverse of the operation described in Eq. 1 to reconstruct the phenotypic measurements ($\hat y$) from any selected subset of background-averaged epistatic terms ($\bar \omega _{{\mathrm{sig}}}$):

2 $$\hat y = {\mathbf{\Omega }}^{ - 1}\bar \omega _{{\mathrm{sig}}},$$

A comparison of $\hat y$, the reconstructed phenotypes, with *y*, the measured phenotypes, indicates the extent to which the epistasis terms included in $\bar \omega _{{\mathrm{sig}}}$ capture the total information contained in all 8192 terms.

**Fig. 3:**
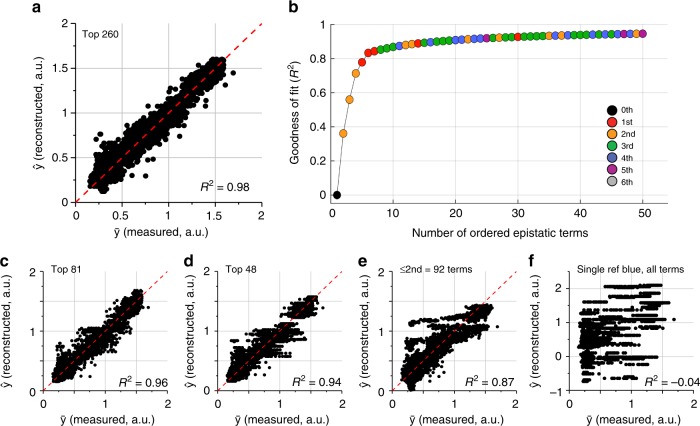
Sparsity in background-averaged (but not single-reference) epistasis. **a** reconstruction of all phenotypes from the 260 significant background averaged epistatic terms displays excellent agreement with measured data (goodness-of-fit *R*^2^ = 0.98, see “Methods”). **b** A plot of goodness-of-fit against number of included epistatic terms arranged by degree of contribution, indicating extraordinary sparsity in information content. Colors show the order of epistasis. **c**, **d** Consistent with sparsity, reconstruction of phenotypes with the top 81 (**c**) or top 48 (**d**) terms shows good agreement with measured data. **e** Reconstruction with only second order terms shows poorer agreement with data despite larger number of included terms, indicating the relevance of higher-order epistasis. **f** Single-reference epistasis shows no predictive power in reconstructing phenotypes, indicating lack of sparsity in this form of epistasis. Noise analysis for single reference epistasis for different genotypes as a reference yields highly variable numbers of significant terms. The plot shows the reconstruction for the parental blue genotype, for which we recover a mere 31 significant terms. Phenotypic reconstruction is poor and exhibits discrete levels due to the low number of epistatic terms

For $\bar \omega _{{\mathrm{sig}}}$ comprising all 260 statistically significant background-averaged epistatic terms, a plot of reconstructed phenotypes ($\hat y$) against actual phenotypes ($\bar y$) shows a goodness of fit coefficient (*R*^2^) of 0.98 (Fig. 3a and “Methods”), demonstrating nearly perfect agreement. This finding means (1) that the experimentally resolvable epistatic terms in fact suffice to represent all phenotypes, and (2) that epistasis is sparse, with only 260 out of 8192 terms (3.2%) capturing close to all of the total information content. An analysis of the contribution of each mutated position to epistasis shows that 11 of the 13 total positions contribute to at least some of the 260 significant terms (Table 1). Thus, sparsity is not a trivial consequence of the irrelevance of many sequence positions. Instead, it says that mutations that contribute to phenotype are involved in much fewer epistatic interactions than theoretically possible.

**Table 1:** Significant epistatic contributions for mutated positions

Position:	1	2	3	4	5	6	7	8	9	10	11	12	13
mTagBFP2 residue:	D20	V45	L63	T127	F143	N158	S168	A172	A174	Y197	N206	N207	K231
Epistatic terms:	44	129	125	88	138	137	63	36	109	124	0	52	0
Frequency (out of 260):	0.16	0.46	0.45	0.32	0.49	0.49	0.23	0.13	0.39	0.44	0	0.19	0

For each of the 13 mutated positions (mTagBFP2 numbering indicated, PDB 3M24), the table shows the number and frequency of contributions made to the 260 statistically significant epistatic terms. The data show that all but two positions contribute to the pattern of epistasis that underlies the path of variation from the blue to red FP variant.

How sparse is epistasis? To examine this, we calculated the goodness of fit between phenotypic data and prediction as a function of the number of included epistatic terms in $\bar \omega _{{\mathrm{sig}}}$, ordered by the size of their contribution to the explanatory power (Fig. 3b). The result demonstrates that background-averaged epistasis is remarkably sparse. Just the top 48 or 81 terms suffice to achieve an *R*^2^ = 0.94 or 0.96, respectively (Fig. 3c, d), indicating that <1% of epistatic terms are enough to specify phenotypes with good accuracy. Interestingly, retaining only epistatic terms up to the second-yields weaker predictive power (*R*^2^ = 0.87) despite including nearly double the number of terms (compare Fig. 3c, d with 3e). Thus, phenotypes can be represented by a very small number of background-averaged epistatic terms, but these range from low to high order.

In contrast, sparse encoding of phenotypes is not evident with the single-reference form of epistasis (Fig. 3f). Taking a particular genotype as a reference for mutational effects, the predictive power of phenotypes based on significant terms is negligible (Fig. 3f, *R*^2^ = −0.04 for the parental blue genotype), and reasonable values for *R*^2^ are only achieved by inclusion of all terms up the 11th order (Supplementary Fig. 8). This indicates essentially no information compression by this approach to epistasis. However, the fact that epistasis is sparse in at least one representation (with background averaging) exposes an important finding—the quantity of information linking genotype to phenotype is fundamentally low relative to theoretical limits.

### Practically learning epistasis—an experimental approach

Background-averaged epistasis provides an efficient, low-dimensional representation of protein phenotype, but this observation does not itself provide a practical solution to identifying the relevant terms. The problem is that background-averaging (at any epistatic order) requires complete knowledge of phenotypes for all combinations of mutants, an impossible proposition in general. So, how do the findings reported above help us to practically learn the relevant epistatic terms? A path forward comes from the finding of sparsity in epistasis. In the field of signal processing, the theory of compressed sensing (CS) states that if a signal displays sparsity in some representation, it is possible to accurately reconstruct the signal from just a limited number of random measurements by employing an optimization procedure that enforces the sparsity in that representation^27^. For proteins, this implies that we should be able to learn the relevant epistatic architecture sampling just a small subset of randomly chosen mutant phenotypes.

**Fig. 4:**
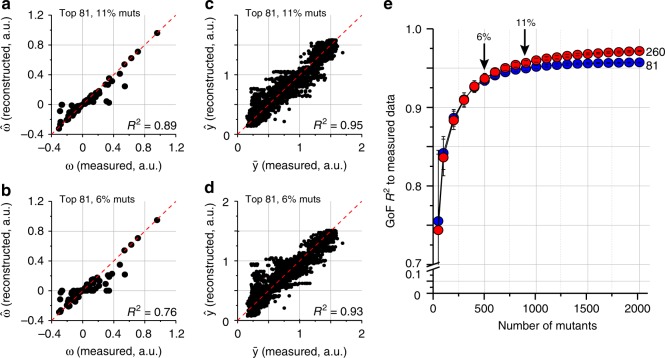
Practical strategies for learning the epistatic structure: experiment. **a**, **b** Estimation of the top epistatic terms (including a broad range of high-order terms) from random samplings of phenotypes for 6–11% of variants, using the method of compressive sensing (CS). **c**, **d** Reconstruction of all phenotypes from the estimated epistatic terms in panels (**a**–**b**). The data show excellent approximation of relevant high-order epistasis and prediction of phenotypes from sparse sampling of data. **e** Goodness of phenotype prediction for all 8192 variants as a function of CS-based estimation of epistasis (top 81 or 260 terms) from many trials of sampling the indicated number of variants

To test this, we developed a simple implementation of the CS algorithm (see “Methods”, and used it to learn the top background-averaged epistatic terms from sparse sampling of the phenotype data. In the CS algorithm, epistatic terms ($\bar \omega$, Eq. 1) are computationally estimated under two mathematical constraints: (1) that they optimally reproduce the randomly selected subset of the data, and (2) that the sum of all epistatic terms (the so-called L1-norm of the epistasis vector) is minimized, the condition that imposes sparsity (see “Methods”). Using this approach, we find that in fact, phenotypes from just a small fraction (~6–11%) of mutants suffice to accurately estimate the top background averaged epistatic terms computed from using data for all mutants (Fig. 4a, b). Not surprisingly, epistatic terms obtained from the L1 minimization can then be used (using Eq. 2) to predict the phenotypes of all variants with excellent accuracy (Fig. 4c, d). A scan over the number of mutants used for prediction shows that the top epistatic terms are asymptotically well-estimated from very modest samplings of mutants (Fig. 4e, and see Supplementary Fig. 9 for less sparse datasets). Note that this procedure does not simply amount to systematically sampling the low-order mutants; instead, the key is to sparsely sample over the space of all mutant combinations—a prescription for experiment design that is apparently best-suited for systems with sparse, high-order epistatic constraints.

In applying this approach for proteins in general, it will be important to understand how the sampling of mutations—the size of the experiment—scales with the number of sequence positions undergoing variation—the size of the problem. Since the latter grows exponentially, it seems likely that the degree of sparsity will be an even greater productive constraint for larger problems.

### Practically learning epistasis—a statistical approach

**Fig. 5:**
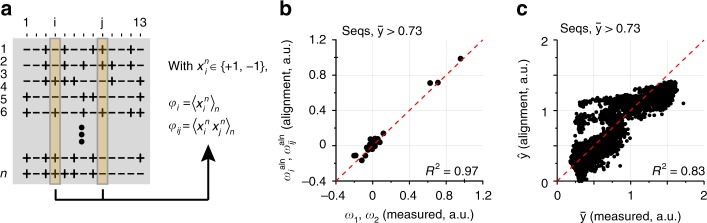
Practical strategies for learning the epistatic structure: sequence statistics. **a** A representation of a multiple sequence alignment (MSA) $x_i^n$ comprising *n* functional sequences (defined as those with *y* > 0.73) by the *i* = (1…13) mutated positions; amino acids are represented by +1 and −1 to indicate residues in mTagBFP2 or mKate2, respectively. From the MSA, we can compute the average value of each position ($\varphi _i = \left\langle {x_i^n} \right\rangle _n$), and the joint expectation of pairs of positions ($\varphi _{ij} = \left\langle {x_i^nx_j^n} \right\rangle _n$). From these we calculate alignment epistatic terms $\omega _i^{{\mathrm{aln}}}$and $\omega _{ij}^{{\mathrm{aln}}}$ as indicated in the “Methods”. **b**, **c** Estimation of measured first and second order epistatic terms (**b**) and consequently, the ability to reconstruct all phenotypes (**c**) using only the first and second alignment statistics

A completely distinct approach for learning the epistatic architecture is suggested by analyzing the statistics of amino acid frequencies in an ensemble of functional sequences. The general idea is that the functional constraints on and between mutated positions should be reflected in the frequencies and correlations of amino acids in sequences that satisfy a threshold of functional selection—a statistical analog of epistatic interactions. To examine this, we used the current data to mimic a collection of functional sequences from the evolutionary record: we constructed a multiple sequence alignment of FP variants with brightness at or above the minimal value of the parental genotypes (mKate2, *y* > 0.73, *n* = 2032 sequences), and computed the statistics of amino acid occurrence and correlations at those positions. Representing the two amino acids *x* at each position *i* with −1 and +1 respectively, we find that the average value over all *n* functional sequences $\left( {\left\langle {x_i^n} \right\rangle _n} \right)$ and the joint expectation between pairs of positions *i* and *j* $\left( {\left\langle {x_i^nx_j^n} \right\rangle _n} \right)$ closely approximate the background averaged first-order and pairwise epistatic terms determined experimentally (up to a known scaling factor; Fig. 5a, b, “Methods”, and ref. ^28^). This relationship holds even with sub-sampling functional sequences included in the alignment (Supplementary Fig. 10). From these alignment-derived epistasis terms, it is again possible to quantitatively predict the phenotypes determined (Fig. 5c) to an extent that approaches what is possible by just limiting epistasis to the second order (Fig. 3e).

This result demonstrates a key conceptual link between epistasis averaged over genetic backgrounds and statistical correlations averaged over sequences displaying function above a threshold, a condition analogous to the process of natural selection. This connection is the fundamental premise of coevolution-based methods that use amino acid correlations in multiple sequence alignments to estimate structural^29–31^ or functional^32–36^ couplings between residues in proteins. Though these methods have provided important insights^37–41^, our findings provide clear evidence that accurate phenotype prediction will generally require knowledge of higher-order epistatic terms as well. Such information is not formally included in current coevolution methods, but may be accessible if alignments are deep enough or the problem of epistasis in full proteins is sparse enough.

### Functional connectivity of the sequence space

How does the pattern of epistasis control the topology of the functional sequence space linking the blue and red variants of eqFP611? Indeed, the existence of severe forms of epistasis (e.g., sign epistasis or reciprocal sign epistasis^4^, in which intermediates along an evolutionary trajectory can fall below the selection threshold) can limit or even abrogate the existence of single-step (or “connected”) paths between functional genotypes^42^. Thus, the study of the structure and connectivity of the space of functional genotypes is important for understanding the relationship of epistasis to evolvability. In our dataset, nearly 50% of the statistically significant pairwise epistatic interactions represent cases of sign or reciprocal-sign epistasis (Supplementary Fig. 11), indicating that functional connectivity of the space linking the parental variants of eqFP611 is hardly guaranteed.

**Fig. 6:**
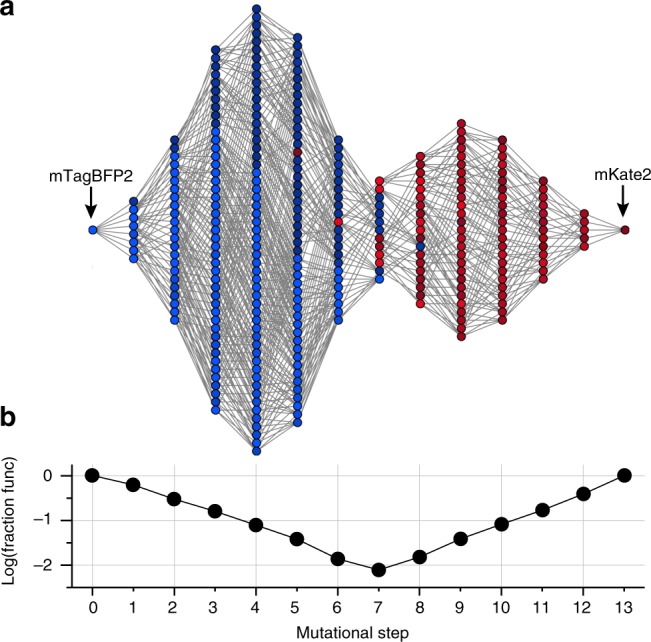
Single-step connectivity of the sequence space linking mTagBFP2 and mKate2. **a** A graph of all genotypes comprising a set of sequences connected by single-step variation, as a function of mutational step from mTagBFP2 to mKate2. The brightness threshold for selection of genotypes is at the level of the mKate2. Thus, the sequence space linking the two parental genotypes is fully connected through single mutations without loss of parental function, and the shape of the solution space involves a thin neck near the middle. **b** The fraction of connected genotypes at each step of mutation reinforces the notion that the space is most constrained at the thin neck, a consequence of severe epistatic constraints at the intermediate steps

Figure 6a shows the network of all functionally connected 13-step paths—the “solution space”—between the blue and red parental variants (*y* > 0.73, the value of the red parent, and see Supplementary Fig. 12). The genotypes are colored according to fluorescence and edges represent single mutations between them. The data show (1) that the sequence space linking the parental genotypes is in fact fully connected at the functional threshold defined by these genotypes, (2) that solution space is shaped like a dumbbell, with two densities of functionally bright sequences near to the parental genotypes connected by a narrow neck, and (3) that the color switches at the neck. The shape of the network reflects the pattern of epistasis. For example, the narrowest part of the solution space occurs in the middle where the number of potential combinatorial mutants is the largest (Fig. 6b), indicating severe epistatic constraints on mutational paths at these steps with regard to retaining brightness along the path (Supplementary Fig. 12D).

Though we focus on single quantity—brightness—as a quantitative phenotype in this work, it is also informative that the fluorescence color switches at the narrow neck. The blue and red spectral states arise from chemically distinct chromophores that are auto-catalytically generated upon folding from amino acids at positions 63–65^43,44^. Interestingly, the data show that L63M—the only chromophore mutation—is necessary but insufficient on its own to produce the red chromophore. Instead, red fluorescence requires several specifically-ordered mutational steps after L63M, another indication of epistasis in the path between the blue and red parental variants.

Overall, the connectivity of the solution space between the blue and red variants shows that the existence of high-order epistatic terms can nevertheless be consistent with evolution through stepwise variation and selection. The effect of epistasis is in specifying the topology of the solution space and in restricting the number of available paths. For example, at the specified brightness threshold, only 1.36 × 10^5^ out of 6.23 × 10^9^ paths between the blue and red parental genotypes (or, ~0.002%) are functionally connected. Single-step connectivity is an advantageous feature for phenotypic evolution through a process of random variation and selection^2^. Thus, connectivity of the solution space represents a possible example of a constraint on protein sequences that arises from not just from the requirement to fold and function, but from the dynamics of the evolutionary process^40,45,46^.

## Discussion

Defining the pattern of epistasis between amino acids is essential for understanding the basic design of proteins. Given the vast theoretical complexity of epistatic interactions between amino acids, it is essential to carry out model experimental studies as a basis for developing practical strategies. Here, we describe a protocol for barcoded combinatorial mutant protein library construction, which, coupled with quantitative high-throughput phenotyping, enables a systematic analysis of high-order epistasis. The data enable a deep quantitative analysis of the extent, pattern, and combinatorial complexity of amino acid interactions within a protein.

For the mutational space linking red and blue variants of the eqFP611 fluorescent protein (a total of 2^13^ genotypes), we find evidence for many statistically significant high-order epistatic interactions. These findings pose a great challenge for understanding proteins in general; no typical experimental workflows collect the kind of data required to analyze high-order epistasis and in any case, the number of experiments grows unmanageably with the number of sequence variations. But, with background-averaging, we find that epistasis is also profoundly sparse, inspiring the use of powerful analytic tools for defining the epistatic architecture through sparse data collection. Indeed, we show that it is possible to computationally deduce the relevant epistatic terms from sampling only a small fraction of total variants that make up the full mutational landscape. These epistatic terms can then be used to predict the phenotype of all variants. Most strategies for studying proteins have focused on low-order mutagenesis^47–51^, but the data presented here suggest that a different experimental approach—limited sampling of combinations of mutations, and sparse reconstruction to deduce the relevant epistatic terms. Combinatorial studies of RNA mutations have been performed^52^, and recently^53^ using the theoretical analysis we presented in ref. ^14^. Although RNA and proteins use a different chemical alphabet, leading to a different type of physical interactions, initial results show that sparsity may be a basic organizing principle in functional RNA as well.

Interestingly, second order background-averaged epistatic terms are also well approximated by the statistical correlations between amino acids in an alignment of functional protein sequences, providing support for yet another approach. Current alignment-based methods for deducing amino acid couplings in proteins either rely on analysis of conserved, collective correlations between positions^32,34^ or on inference of direct pairwise interactions through inverse methods in statistical physics^29,31,54^. The data presented here provide a critical benchmark for these approaches, defining the minimal epistatic terms that must be estimated in order to successfully relate genotype to phenotype. The sparsity of epistasis may also provide a productive constraint for developing an improved theoretical framework for using statistical coevolution to quantitatively predict protein phenotypes.

It is important to point out that this work represents a focused study of high-order epistasis in a limited number of positions in one model system. Its findings will need to be extended theoretically and tested for generality. Additionally, observable traits will exhibit overall nonlinearities, for example, due to a limited linear range of the measurement or to a saturating organismal fitness^21–23,55^. It is therefore crucial for any study on epistasis to define the quantity for which the analysis is performed. Here we sought to minimize trivial non-linearities using a mechanistically unbiased linear-nonlinear optimization^21,22^ (“Methods”), hence defining our dataset. Further analysis focusing on the potential effect of the lower detection limit in our FACS-seq assay—based on data reconstruction without assigning explicit numeric values to data points below that limit—suggests that the major epistatic contributions are imposed by data within the linear regime of the assay (Supplementary Fig. 13). Whether this holds true in other cases should be carefully monitored.

The data collected here represent a starting point for guiding the next developments, and most importantly, demonstrates the existence and application of sparsity in epistasis to make practical solutions possible.

Knowledge about the type and extent of epistasis, informs us on the number of viable evolutionary trajectories, and hence on both on the potential^42,56^ and repeatability^3,57,58^ of natural evolution. In directed evolution efforts, improved predictive power for unobserved phenotypes may help select evolvable initial templates—with a high fraction of active genotypes in their mutational neighborhood—, potentially in combination with computational protein design algorithms used to identify templates that can bind specific molecules^59–61^.

The identification of highly-epistatic regions^14^ may guide targeted mutagenesis approaches: focusing mutations to epistatic regions may open up adaptive trajectories that otherwise are statistically too unlikely. Furthermore, mapping of higher-order epistasis can identify cooperative units that serve as recombination fragments in knowledge-based DNA shuffling efforts, in a fashion analogous to SCHEMA^13,62,63^, but without the need for three-dimensional protein structural data. Relevant to all the above applications is the ability to rapidly assess a system’s complexity by explorative, not necessarily exhaustive, combinatorial mutagenesis. This, in turn, provides an estimate for the number of phenotypic measurements that is necessary for an acceptably accurate parametrization of the system, and therefore directly informs our choice for an adequately high-throughput experimental assay.

The current work focused on epistasis between amino acid substitutions in proteins, but its conceptual framework is also well-suited to quantify interactions among genes^64–67^ in cellular pathways—which in fact is closer to the original definition of epistasis^68,69^. Data acquired in combinatorial mutant screens^70^ or combinatorial knockout experiments^71^ would provide the basis to empirically tackle the high-dimensional nature of complex genetic diseases^72,73^.

Eventually, what controls the prevalence, distribution, and spatial architecture of epistasis in proteins? Why should it be sparse? Part of the explanation comes from physical considerations; for example, the forces that bind atoms mostly act locally in protein structures, a property that requires long-range epistatic terms to be built up from the coupling of local interactions. However, the finding that the blue and red variants of eqFP611 are connected by single-step mutations suggests the possibility of other constraints as well: if evolution is facilitated by the stepwise functional connectivity of genotypes, then it is clear that any pattern of internal epistasis that is inconsistent with such connectivity will be less fit under varying selective conditions. The practical analysis of epistasis is the starting point for testing such ideas, and this work provides a path towards that goal.

## Methods

### Combinatorial library construction

The combinatorial library of 2^13^ FP variants was constructed by an iterative synthesis protocol in which mutant combinations and an associated barcode are co-assembled in a derivative of the pRD007 plasmid^74^ (Supplementary Fig. 1). Briefly, 34 DNA segments were synthesized (500 bp gBlocks, IDT Inc), each comprising a portion of the FP coding sequence, a 5′ barcode encoding the identity of mutations within this region, and three restriction sites in between (a Type II site flanked by two non-palindromic Type IIs sites). Sequences were optimized to avoid AGA and AGG codons, which are rare in *E. coli*. Barcodes are designed to have Hamming distance of at least two between each other, with each segment barcode comprising three bases plus a parity base (which represents the numeric sum of the three bases modulo four). The Type IIs sites permit scarless in-frame joining of segments by cutting outside of the recognition sequence and the Type II sites increases cloning efficiency by elimination of uncut or back-ligated species. Each segment encodes one to three mutated positions, with the most 5′ segment of the FP gene including an IPTG-inducible promoter from pTrc99A^75^ and a random 12 base pair “uniqueness” barcode that uniquely labels each individual clone. The FP genes are constructed iteratively 3′ to 5′, where at each step, one segment is ligated into the host vector, transformed into *Escherichia coli* DH5*α*^76^, grown overnight, and the resulting plasmid library isolated to serve the host vector for the next round. In this process, combinations of mutants and associated segment barcodes are assembled together. A key technique is the alternating use of two sets of type IIS and Type II restriction endonucleases (Supplementary Fig. 1). After complete assembly, the library is transformed into *E. coli* MC1061^77^ (AVB100, Avidity Inc), at low DNA concentration (5 ng DNA total) to suppress multiple transformants (typical library size of 5 *×* 10^6^) and bottlenecked to 1 *×* 10^6^ individual transformants to ensure high enough multiplicity of uniqueness barcodes. Strain MC1061 was chosen for robust growth and to avoid issues that arise with regular cloning strains, such as filamentation, which would increase noise in the per cell fluorescence brightness determination. Sequences for all construction segments and plasmids are provided in Supplementary Data 1 and 2.

### Cell sorting

MC1061 cells containing the FP library were grown at 37 °C to an optical density of 0.8 in LB plus 50 µg/ml kanamycin, induced with 200 μM IPTG for one hour, and kept at 16 °C overnight. Cells are then washed and resuspended in deionized sterile water, diluted to ~10^7^/ml, and sorted on a BD facsaria (UT Southwestern Medical Center cytometry core) at excitation/emission wavelengths of 405/455 nm and 532/610 nm. The total sorted population was 6.2 × 10^7^ cells, and gating thresholds were chosen to recover the top 1% of this population in each channel, concurrently with forward-scatter gating consistent with live cell size. Fluorescence gating by threshold yields a graded output because single cells encoding any particular allele exhibit fluorescence that follows a near log-normal count distribution (see e.g., ref. ^49^ and Supplementary Fig. 2). Screening of colonies on plates yielded no evidence for multi-colored proteins (Supplementary Fig. 3), justifying stringent sorting thresholds along the observed phenotypic axes. Sorted cells were recovered in LB medium without antibiotics, grown overnight in LB plus 50 µg/ml kanamycin, and subject to plasmid isolation for deep sequencing.

### Sequencing, error correction, and phenotype determination

Samples for sequencing were prepared by PCR from plasmid libraries before or after selection using primers that incorporate Illumina adaptor sequences, a barcode specifying origin (unsorted input or sorted output color channel), and a random stretch of five nucleotides in the initial 5′ region for phasing and cluster definition. Products were pooled in a ratio of 10:1:2 representing input, red, and blue output channels, and paired-end PE-100 sequencing was performed on a single lane of an Illumina Genome Analyzer IIx (UT Southwestern genomics core). Raw FASTQ files from the Illumina base-caller were processed with custom scripts in unix and matlab, and subjected to stringent quality filtering involving three criteria: 100% correct reads within a mask around the barcodes, correct specific barcodes, and a Q-score of at least 30 for each nucleotide in the random “uniqueness” barcode. Primer sequences and scripts are provided as Supplementary Data 1 and Supplementary Software 1. Sequencing reads have been deposited in the Sequencing Reads Archive.

The uniqueness barcode provides a mechanism to correct for mis-sorting events and unobserved spurious mutations that can introduce errors in assigning phenotypes. For each allele-specific barcode *a* we compute input and output counts for each uniqueness barcode *k* as $N_{a,k}^{{\mathrm{in}}}$ and $N_{a,k}^{{\mathrm{out}}}$, and a linear allelic enrichment $E_a = \mathop {\sum }\limits_k N_{a,k}^{{\mathrm{out}}}/N_{a,k}^{{\mathrm{in}}}$. Modeling experimental errors as a Poisson-process, we compute noise on the output counts of a uniqueness barcode as $\langle {\mathrm{noise}} \rangle_{i} \propto \root {\beta } \of {{N_{a,k}^{{\mathrm{out}}}}} \propto \root {\beta } \of {{E_aN_{a,k}^{{\mathrm{in}}}}}$, calculate the Z scores for the individual uniqueness bars as

3 $${\cal{Z}} = \frac{{N_{a,k}^{\mathrm{out}} - E_aN_{a,k}^{\mathrm{in}}}}{{\root {\beta } \of {{E_aN_{a,k}^{\mathrm{in}}}}}},$$

and set the upper and lower boundaries for inclusion in the data per uniqueness barcode as ${\cal{L}}_{{\mathrm{upper}}} = c_1{\cal{Z}}$ and ${\cal{L}}_{{\mathrm{lower}}} = c_2{\cal{Z}}$. Choices of parameters (*β* = 1*/*0.35, *c*_1_ = 35, and *c*_2_ = 15) were based on robustness across color channels and for alleles over the full range of enrichments *E*_*a*_ (Supplementary Fig. 4). Three rounds of outlier rejection led to removal of 2% of counts, after which final enrichments were calculated.

The values for the linear enrichments *E*_*a*_ after correction were then normalized by the known brightness ratio of 25.0 × 10^3^/32.4 × 10^3^ = 0.772 between the red and the blue parental genotypes^19,20^ (see also www.fpbase.org), and taking into account that the red fluorescence is assayed here at wavelengths different from its excitation and emission peaks, which gives it a lower apparent brightness in our assay by a factor 0.22 (calculated from the measured spectral data). The expected brightness ratio for the parental blue and red genotypes becomes 0.772 × 0.222 = 0.172, and enrichment values in the two color channels are normalized to reflect this ratio. Subsequently, the normalized enrichment values from the red and blue color channel for each allele *a* are combined quadratically according to $x_a = \sqrt {(E_{a,{\mathrm{blue}}})^2 + (E_{a,{\mathrm{red}}})^2}$.

Analysis of epistasis requires elimination of trivial global nonlinearities in the data that arise from the experimental or analytic process^22^. The general principle is that trivial nonlinearities will systematically influence every variant, while nonlinearities due to intramolecular epistasis are highly specific properties of a few variants. A logical approach is to find the simplest empirical transform $\bar y = f(\bar x)$ that minimizes the global non-linearity, especially in the most well determined (i.e., low-order) terms^21^. Practically, we maximize *R*^2^ between the measured data and data reconstructed with low order epistatic terms only, which is done by minimizing $\left\| {f\left( {\bar x} \right) - \left[ {\left( {\bf{\Omega }} \right)^{ - 1}{\mathbf{S}}{\bf{\Omega }}} \right]f\left( {\bar x} \right)} \right\|_2^2{\mathrm{/var}}\left( {f\left( {\bar x} \right)} \right)$, where **Ω** is the epistasis operator and **S** is a matrix which selects only epistatic terms up to order two. This led to $f\left( {\bar x} \right) = \bar x^\alpha$ with *α* = 0.44. Upon transformation, the data corresponding to genotypes with zero brightness are regularized by adding pseudocounts based on fitting noise present in non-functional genotypes, resulting in the vector of final brightness phenotypes $({\bar{\mathrm{y}}})$ for all variants. Supplementary Fig. 6 shows that the conclusions in this work are robust to these data processing steps. matlab scripts for all data processing steps are provided as Supplementary Software 1. A replicate experiment was performed starting from the same bottlenecked population, sorted and sequenced independently (on a MiSeq platform) and analyzed as indicated above (Supplementary Fig. 7).

### Analysis of epistasis

For a single amino acid substitution at each of *N* positions, the full space of possible genotypes corresponds to 2^*N*^ individual variants. With phenotypes for all variants (${\bar{\mathrm{y}}}$) in a form where independence corresponds to additivity of mutation effects, the analysis of epistasis corresponds to a linear mapping $\bar \omega = {\mathbf{\Omega }}\bar y$, where $\bar \omega$ is the vector with epistatic terms of all orders (0 to *N*), and **Ω** is the epistasis operator^14^. For background-averaged epistasis, **Ω** is a weighted Walsh-Hadamard transform^78^, a class of generalized Fourier transforms^79^, and can be written as **Ω** = **VH** where **V** is a weighting matrix and **H** is the Hadamard matrix, both of which can be recursively defined:

$${\mathbf{V}}_{n + 1} = \left( {\begin{array}{*{20}{c}} {\frac{1}{2}{\mathbf{V}}_n} & 0 \\ 0 & { - {\mathbf{V}}_n} \end{array}} \right){\mathrm{and}}\;{\mathbf{H}}_{n + 1} = \left( {\begin{array}{*{20}{c}} {{\mathbf{H}}_n} & {{\mathbf{H}}_n} \\ {{\mathbf{H}}_n} & { - {\mathbf{H}}_n} \end{array}} \right),$$

with **V**_0_ = **H**_0_ = 1, and *n* = {0…*N*–1}. For standard, single-reference epistasis, **Ω** = **VX**^*T*^**H**, where

$${\mathbf{X}}_{n + 1} = \left( {\begin{array}{*{20}{c}} {{\mathbf{X}}_n} & 0 \\ {{\mathbf{X}}_n} & {{\mathbf{X}}_n} \end{array}} \right),$$

with ${\mathbf{X}}_0 = 1$. Conceptually, standard epistasis represents a local (Taylor) approximation of the fitness landscape expanded around one reference genotype, while background-averaged epistasis approximates the landscape in terms of its global features over the space of all possible genotypes. See ref. ^14^ for a more extensive description of the theory.

### Functional trajectories and genotypic connectivity

To obtain the number of functional single-step trajectories, we compute an adjacency matrix between functional genotypes by binarization of the full genotype adjacency matrix above a select threshold brightness (for Fig. 6, *y* = 0.73, the value for the red parental variant). From the binarized adjacency matrix, the (*i*,*j*)-th element of the *m*th power of the matrix gives the number of functional *m*-step trajectories that exist between genotypes *i* and *j*. Summing over the powers of this matrix to any order *M* gives all viable trajectories consisting of *M* or fewer steps in the sequence space. Conversion of the resulting summed matrix to block-diagonal form produces a “genotypic connectogram”—a graph that directly reveals the connectivity and topology of viable genotypes (Supplementary Fig. 12C).

### Sparse optimization and phenotype reconstruction

CS is performed by finding a sparse representation for the data on the basis of a (small) subset of measurements, and subsequent reconstruction the full dataset by inverse transformation from the sparse representation back to the data domain (see ref. ^27^ for an excellent presentation of the theory). Finding the optimal sparse distribution of epistatic terms is achieved by L1-norm minimization and was performed in this study in matlab using the yall1 solver, version 1.4^80^. The performance for mutant prediction is scored by the Goodness of Prediction (GoP) parameter $\frac{1}{{1 + {\mathrm{SSE}}/{\mathrm{SST}}}}$, where SSE is the sum of squared errors between reconstruction phenotypes and the measured values, and SST is the total sum of squares. matlab scripts are provided as Supplementary Software 1.

### Alignment epistasis

First-order alignment epistasis is calculated according to $\omega _i^{{\mathrm{aln}}} = \varphi _i\frac{{2N_{{\mathrm{tot}}}^{{\mathrm{func}}}}}{{N_{{\mathrm{tot}}}}}$, where *φ*_*i*_ is the average value $\left( {\left\langle {x_i^n} \right\rangle _n} \right)$ of the two states of amino acid *x* at position *i* represented as −1 and +1, over the *n* functional sequences in the alignment. $N_{{\mathrm{tot}}}^{{\mathrm{func}}}$ and *N*_tot_ are the number of functional sequences and the total number of sequences in the combinatorial dataset (see ref. ^28^ for extended theory). Second order alignment epistasis is calculated according to $\omega _{ij}^{{\mathrm{aln}}} = \varphi _{ij}\frac{{4N_{{\mathrm{tot}}}^{{\mathrm{func}}}}}{{N_{{\mathrm{tot}}}}}$, where *φ*_*ij*_ is the joint expectation between pairs of positions *i* and *j* $\left( {\left\langle {x_i^nx_j^n} \right\rangle _n} \right)$. In the current dataset we have explicit numbers for $N_{{\mathrm{tot}}}^{{\mathrm{func}}}$ and *N*_tot_ and therefore we can numerically compare this with calculated background-averaged epistatic terms (Fig. 5 and Supplementary Fig. 10).

### Reporting summary

Further information on research design is available in the Nature Research Reporting Summary linked to this article.

## Supplementary information

Supplementary Information

Reporting Summary

Description of Additional Supplementary Files

Supplementary Software 1

Supplementary Data 1

Supplementary Data 2

Supplementary Data 3

Supplementary Data 4

Supplementary Data 5

## Data Availability

A detailed construction overview for the mutant library, including used DNA fragments and primers, is part of the Supplementary Information. Raw sequencing reads analyzed in this study have been deposited in the Sequencing Reads Archive under BioProject number PRJNA560590. All other data generated or analyzed in this study are included in this published article (and in its accompanying Supplementary Information).

## Code availability

All code used in this study is included as Supplementary Software 1. The computational scripts will reproduce the figures in the article.
