# Supplementary material for: Learning the pattern of epistasis linking genotype and phenotype in a protein
Source: Nat Commun. 2019 Sep 16;10:4213. doi: 10.1038/s41467-019-12130-8 (PMC6746860; doi:10.1038/s41467-019-12130-8)
Supplement: Supplementary file 1 — Supplementary Information [file 41467_2019_12130_MOESM1_ESM.pdf]

Supplementary Information

Learning the pattern of epistasis linking genotype and phenotype in a protein

Frank J. Poelwijk, Michael Socolich, and Rama Ranganathan

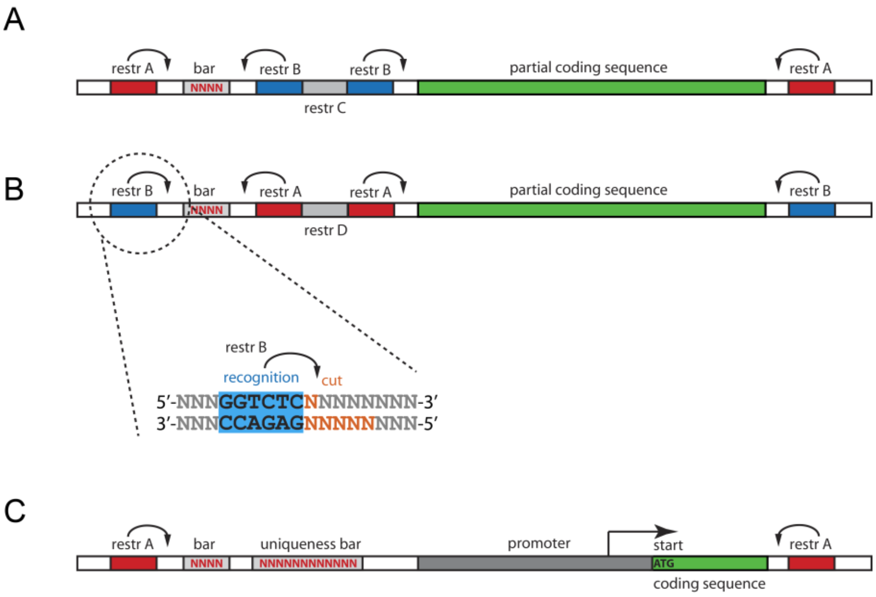

Supplementary Figure 1: Combinatorial library synthesis. The library of FP variants is generated by sequential restriction and ligation steps incorporating pre-synthesized DNA segments (gBlock, IDT Inc.) from 3' to 5' that each contain a subset of the mutated positions and a partial barcode that indicates each combination of mutations. The number of segments is determined by a trade-off between minimizing the number of assembly steps and minimizing cost; here 34 construction segments were used – segments 1-4 with three mutations each (**A-B**) and a final fifth segment created by PCR with one mutable position and the promoter (**C**). Segments 1-4 have a design that alternates between the schemes shown in panels A and B, explained below. The barcodes are designed to have minimal Hamming distance of two between each other, and are embedded in a flanking sequence that is designed to avoid palindromes, long repeats, or restriction sites used in construction. The gene assembly process is as follows: The first construction segment is cut with Type IIS restriction enzyme BsrDI (restr A), and ligated to target vector pFPH, a derivative of pRD007[1]. After transformation and isolation of plasmid DNA, the resulting population of plasmids and the second segment are cut together with Type IIS enzyme BsaI (restr B), purified and ligated, inserting the second segment 5' to the first and juxtaposing the segment barcodes. A "kill cut" is made with PstI (restr C) to reduce propagation of uncut or back-ligated species, and the ligation reaction is transformed and DNA isolated. This procedure is repeated for the remaining segments, alternating the use of the restriction enzymes as per panels A and B (restr D is NdeI). The final step incorporates the 12 bases long random "uniqueness" barcode, present in the oligonucleotide primer that creates this fragment.

Care was indeed taken to insert bar codes at sufficient distance from sequence features impacting the expression of the fluorescent proteins, so that bar code variation does not by itself influences the determination of fitness. An advantage of the combinatorial synthesis approach developed here is that the distance between bar codes and the gene of interest can in principle be chosen to be arbitrarily large. In the plasmid constructed here, the widely-used hybrid trp-lac promoter region (*Ptac*[2]) was incorporated in its unchanged form up to position -172 from the translational start, and the bar codes were placed upstream of this region. As a comparison, for the wild-type *E coli lac* operator the influence of sequence variation dwindles to zero at about position -65 from the transcriptional start[3] (for CAP inactive, which is comparable to *Ptac*). Thus, our bar codes are more than 100 bases away from the point where Kinney *et al.* could not anymore observe influence of sequence variation on expression, and we expect the bar code variation to be fully neutral with respect to fitness.

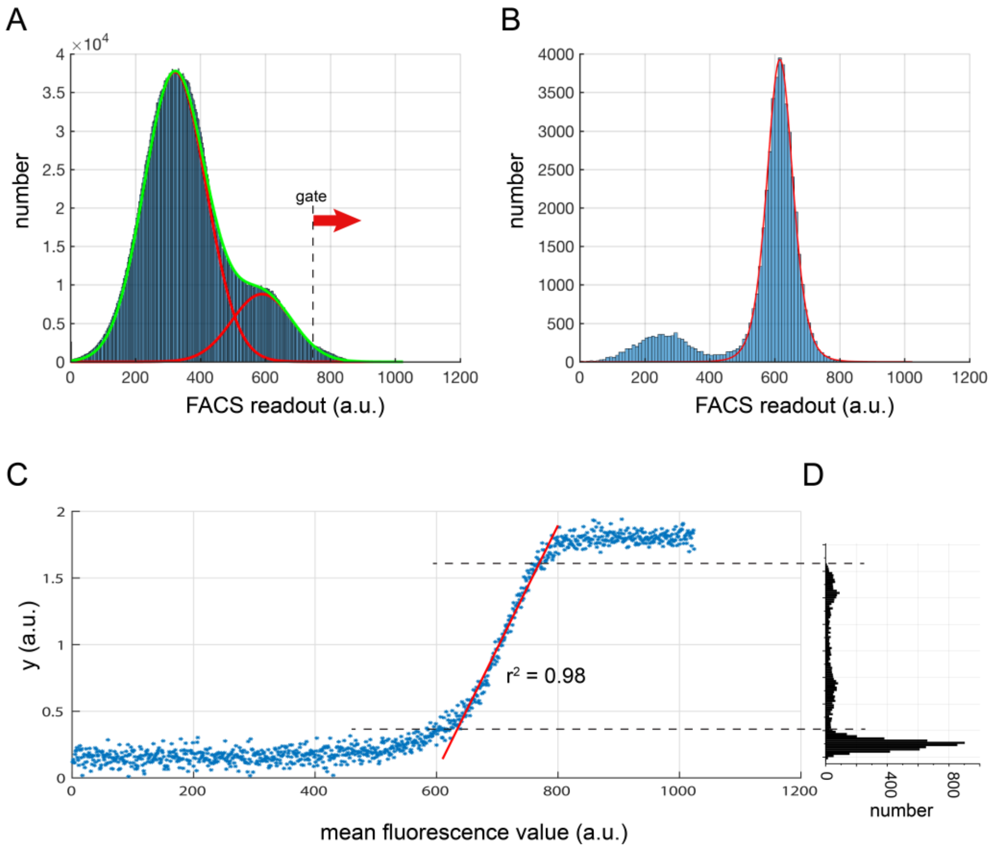

Supplementary Figure 2: Analysis of the FACS-seq assay. **A**, Raw FACS data, showing the distribution of measured fluorescence brightness over the full library of 2^{13} variants in the blue channel. The distribution is bimodal, with variants showing low fluorescence in this channel mixed with a distinct group of more brightly fluorescent variants. The gate for sorting in both red and blue channels is at the top 1% of the empirical distribution (Methods). **B**, A typical fluorescence distribution for a single variant, demonstrating a broad spread over the range of bright phenotypes. The broadness of the distribution enables the frequency of observing variants above the gate threshold to be a smoothly graded quantity. **C**, A simulated mapping between mean fluorescence for individual variants (the "brightness", x-axis) against the phenotype ($\bar{y}$), where the calculation of $\bar{y}$ includes the linear-nonlinear correction for global non-linearities[4] and takes into account typical measurement noise. Assuming fluorescence distributions for individual alleles with the same empirical shape as in panel B, and setting the gate so that 1% of all mutants will pass (level obtained from panel A), the simulation shows a near-linear relationship (indicated by fit, in red) between brightness and the phenotype $\bar{y}$ over the full range of variants with discernable fluorescence in either channel. **D**, phenotype $\bar{y}$ as in Figure 1C, here in relation to fluorescence counts). Note that the saturation regime above the upper dashed line (associated with a brightness much higher than the gate level) can be freely explored computationally by simulating variants with arbitrarily high brightness. However, in the experiment these variants do not occur, simply because the gate is set near the top of the observed brightnesses. In other words, unless the distribution of mutation effects is highly skewed, the experimental gating procedure relative to the maximum brightness will prevent saturation at the higher end.

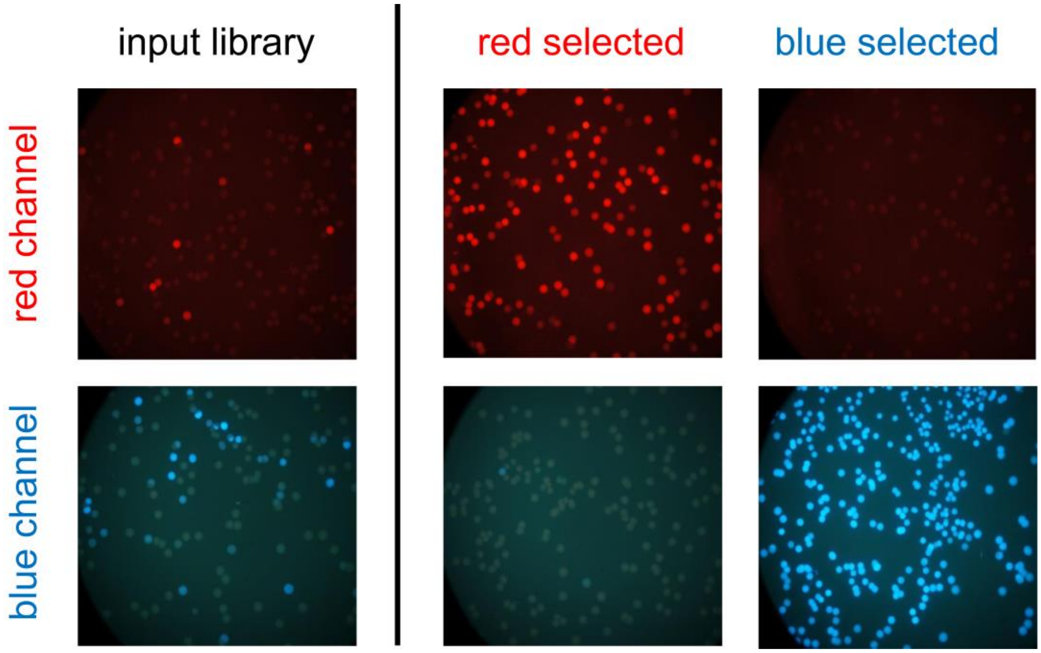

Supplementary Figure 3: Mutant phenotypes pre- and post-sorting. Colonies of a library sample pre-sorting (left column) and post-sorting (right two columns) are imaged in two color channels (excitation 405nm and 532nm respectively, emission recorded using long-pass filters in front of a camera). This manual inspection illustrates the effectiveness of the cell sorting.

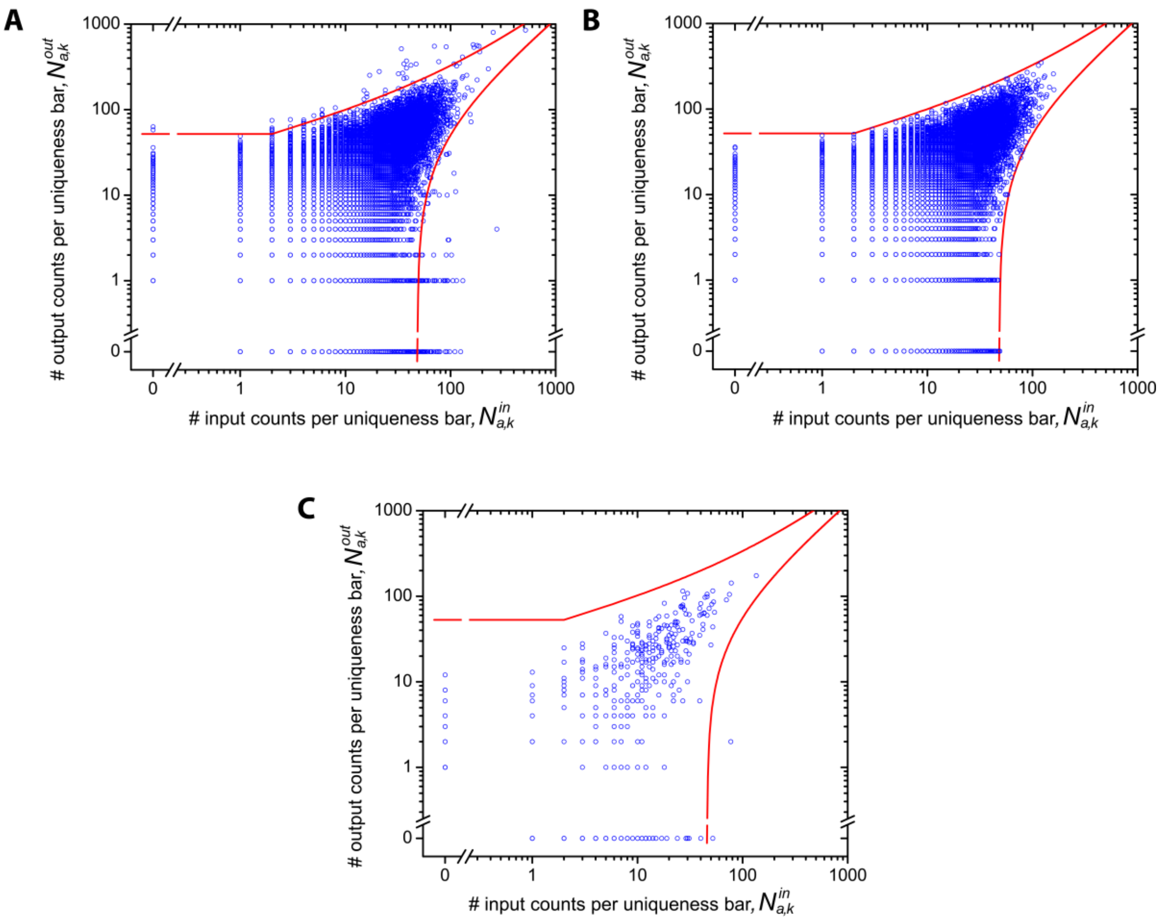

Supplementary Figure 4: Error-correction by outlier suppression. The error correction is described in detail in the Methods section in the main text. **A,** Input and output counts per uniqueness barcode for a set of blue alleles with a mean output count to input count ratio between 1.30 and 1.38. The alleles are pooled here in order to demonstrate the shape of the data cloud with respect to the upper and lower boundaries $\mathcal{L}_{\text{upper}}$ and $\mathcal{L}_{\text{lower}}$ (in red). Shapes of boundaries result from considering counting noise in the experimental approach (see equations in Methods); values for $\mathcal{L}_{\text{upper}}$ and $\mathcal{L}_{\text{lower}}$ are chosen by visual inspection to include the combined data cloud for a large number of alleles with similar count ratio. This results in a mild correction, only for count ratios that deviate from the mean by a large margin. Note that the actual outlier suppression is performed per individual allele, and the numeric values for $\mathcal{L}_{\text{upper}}$ and $\mathcal{L}_{\text{lower}}$ remain the same for the entire dataset, independent of the allele's count ratio or its color. **B,** Counts for the same pooled set of alleles after three iterations of error correction, illustrating which uniqueness bar counts are removed. **C,** Example for an individual allele: raw counts for the wild-type blue allele (allele 1). Two uniqueness barcodes on the right hand side fall outside the boundaries and will be removed from the dataset.

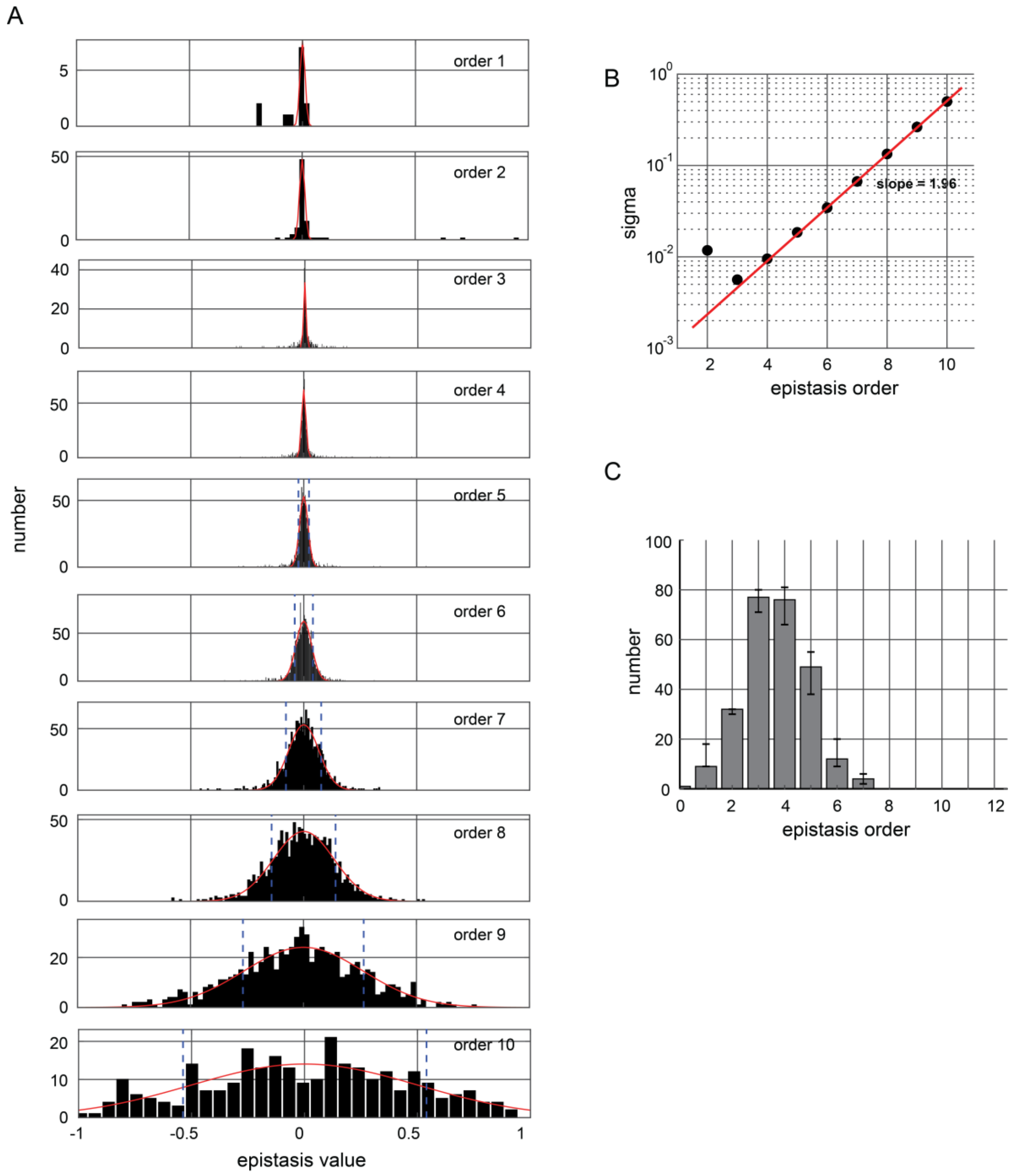

Supplementary Figure 5: Error propagation and determination of significant epistatic terms. **A**, Histograms of calculated epistatic terms for orders 1-10, fitted to Gaussian distributions (in red). The data show that as expected by the propagation of error rule, the variance in the distribution grows with epistatic order. **B**, the logarithm of the fitted Gaussian widths of the histograms as a function of epistatic order. The slope indicates an increase in observed noise of a factor 1.96 per order, close to the theoretical expectation of 2 ($r^2 = 0.99$). The y-axis intercept is at 6.1 x 10-4, suggesting an average per data point error of $6.1 \times 10^{-4} * \sqrt{2^{13}} = 0.055$. C, Number of epistatic terms as a function of order at a significance threshold of $p = 0.01$, after Bonferroni- Šidák correction (as in Fig. 2E). Here, "error bars" indicate the robustness of this distribution to the choice of p-value. The low-range is $p = 0.001$, and the high-range is $p = 0.05$.

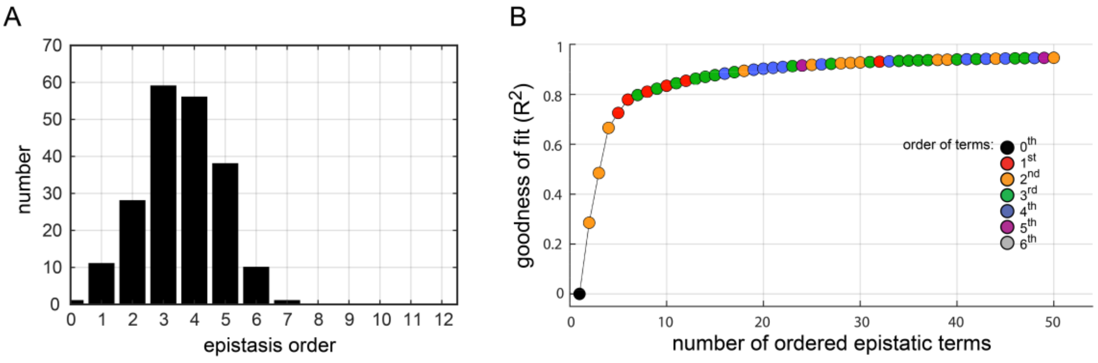

Supplementary Figure 6: Epistasis analysis without removal of global non-linearities. **A**, Histogram of epistatic terms as a function of order at a significance threshold of $p < .01$ (after Bonferroni-Šidák correction for multiple testing), computed exactly as in Fig. 2E, but without the linear-nonlinear transform to minimize global non-linearity in the data. **B**, the Goodness-of-fit between measured and reconstructed phenotypes as a function of number of included epistatic terms, ordered by degree of contribution (analogous to Fig. 3B). The analysis shows that the basic conclusions of this work are not strongly dependent on the nature of the phenotype transform.

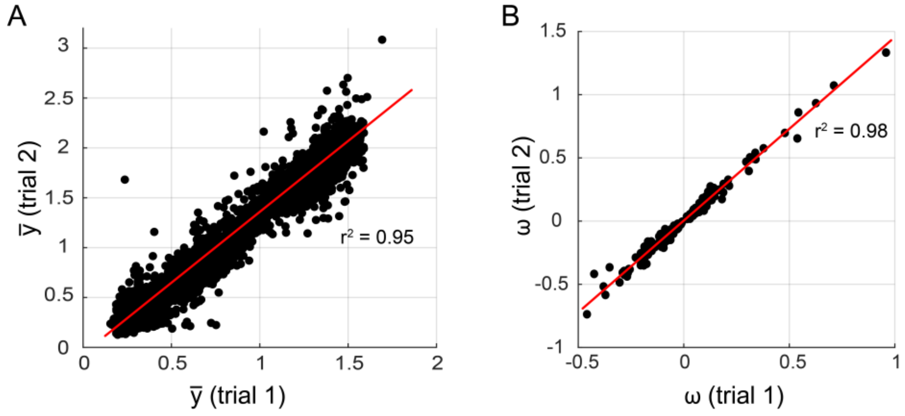

Supplementary Figure 7: Reproducibility of phenotypes and epistasis. **A**, Phenotypes of all 2^{13} FP variants over two independent experimental trials of the FACS-seq experiment. Trial 2 was started from the same bottlenecked population, sorted and sequenced independently and analyzed as indicated in Fig. 1B. The data points exhibit a slope different from one, indicating that the overall ratio between input and output counts differs, possibly due to differences in sample preparation. This multiplication factor has no consequences for the observed prevalence of epistasis, as can be seen in panel B. **B**, The top 260 background-averaged epistatic terms computed for phenotype data from both trials, showing excellent match, again up to a multiplication factor. This indicates the insensitivity of the major background averaged epistatic terms to noise, which is also discussed in ref. 5.

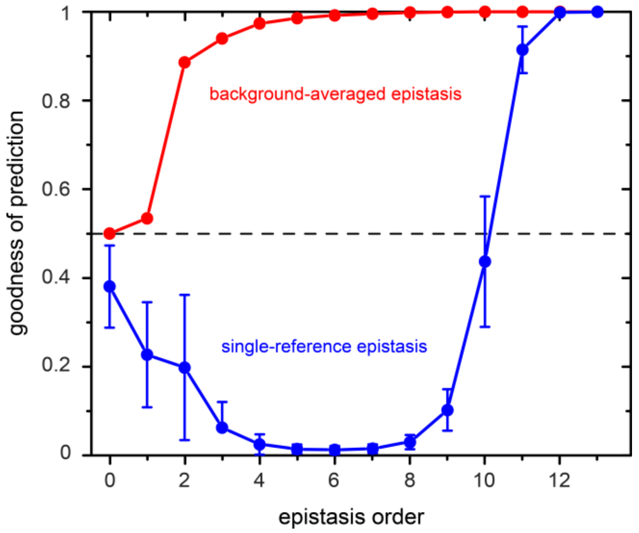

Supplementary Figure 8: Phenotype prediction, a comparison of background-averaged and single-reference epistasis. In this work, we described two formalisms for representing the non-independence of mutations: (1) single-reference epistasis, in which one genotype is selected as the reference for all mutational effects and (2) background-averaged epistasis, in which mutational effects are averaged over the full space of possible variants. Previous work has pointed out the mathematical distinctions[6-9], but the data presented here provide an opportunity to more intuitively understand these formalisms. Both single-reference and background-averaged epistasis transform the same data (phenotypes of variants) into a representation of interactions between the underlying mutations. Thus, they must contain the same total information content, and indeed, can be mathematically interchanged[6]. The distinction between the two is not in the total quantity of information encoded, but in the encoding of that information. Background-averaging produces a sparse representation with most of the phenotypic information compressed in a few top terms, while single-reference epistasis spreads the information over many terms. It is interesting to note that the typical mutant cycle experiment in biochemistry represents an instance of single-reference epistasis in which mutations are seen as perturbations to a "wild-type" state. For this figure the goodness of prediction (GoP, see Methods) for epistatic terms computed using background averaging (red) or with taking a single genotype as a reference (blue, shown is the mean and standard deviation for 100 randomly chosen reference genotypes). A goodness of prediction of 0.5 is expected for a random (fully uninformed) prediction. The data show that prediction using single-reference epistasis only out-performs a uninformed prediction when terms up to the 11th order are included (note that the GoP necessarily converges to unity when all orders of epistasis are included. Thus, epistasis is not sparse when using single-reference definitions.

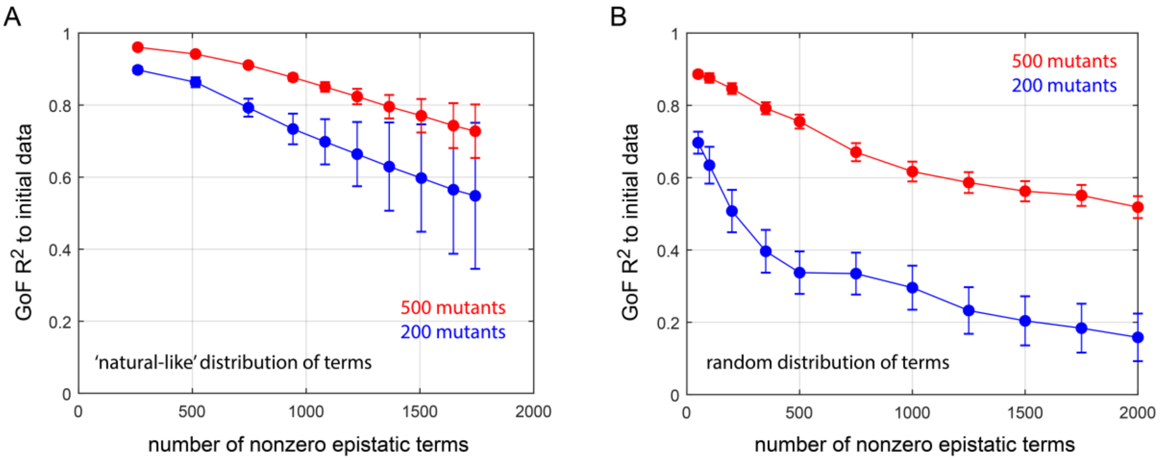

Supplementary Figure 9: Compressed sensing with less sparse datasets. Here we performed the compressed sensing approach with artificially decreased sparsity (more nonzero epistatic terms) in the dataset. **A,** The current dataset is computationally de-sparsified by the following procedure: 1) starting with the 260 significant epistatic terms (other terms set to zero), then 2) per order, randomly choosing *x* terms among the zero terms, where *x* is the number of significant terms of that order among the initial 260 terms (for example, *x* is 76 terms for the fourth order), 3) replacing those zero terms by a value randomly chosen from the already existing values of that order. If no nonzero terms remain at a certain order, no terms are added. In this way the less sparse datasets retain some structure in common with the original dataset. Shown are goodness-of fit between phenotypes of the created datasets and the predictions from the compressed sensing approach, using random subsets of 200 or 500 mutants. The graphs indicate that the approach yields less accurate predictions for less sparse datasets. In fact, theory from compressed sensing, ref. 10, says that in the absence of noise a perfect reconstruction can be achieved by making 3 *s* measurements, where *s* represents the sparsity in the data. Error bars represent standard deviations for 100 independent iterations of the compressed sensing algorithm. **B,** The same compressed sensing approach is performed using simpler artificial random datasets, in which nonzero terms have been added randomly from a uniform distribution between −1 and 1, again using random subsets of 200 or 500 mutants. The decrease in performance for increasingly less sparse datasets is more pronounced than in the datasets in panel A.

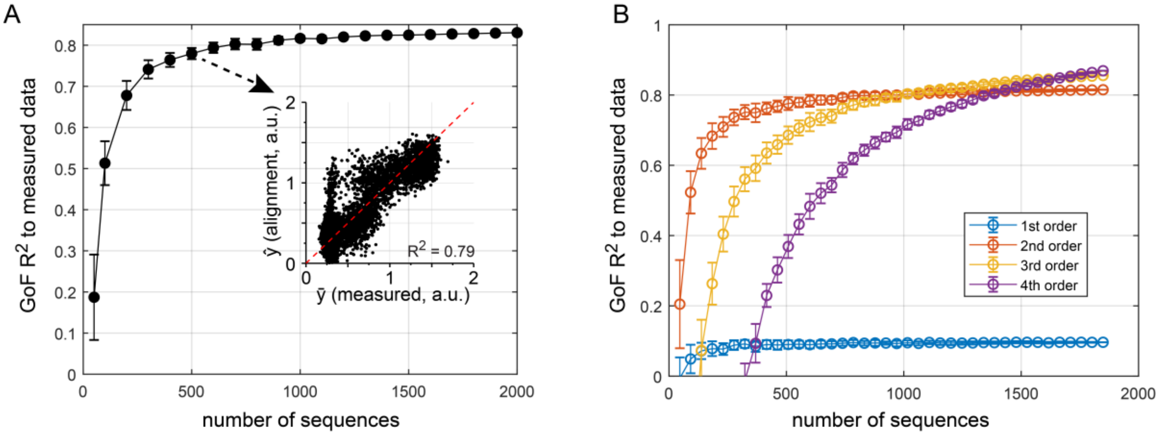

Supplementary Figure 10: Phenotype prediction from alignment statistics, as a function of sequence sampling. **A,** The graph shows the goodness-of-fit R^2 between measured and reconstructed data (as in Fig. 5C) for epistatic terms estimated from alignments of functional sequences sampled from the full alignment of functional sequences (defined as those with $y > 0.73$). The data show rapid convergence of phenotype prediction with even sub-sampling of functional sequences. The inset shows the quality of phenotype reconstruction for one instance of sampling 500 sequences from the full set of functional genotypes (compare with Fig. 5C). **B,** Using different orders of alignment epistasis to predict phenotypes, we observe that 1) first order statistics is not good enough to make meaningful predictions, and 2) even though including higher than second-order terms in principle should improve the accuracy of the predictions, for small alignments of functional genotypes sampling noise will dominate and predictions will actually be worse. Once a substantial number of mutants are included, higher order predictions will indeed be better than predictions using lower orders.

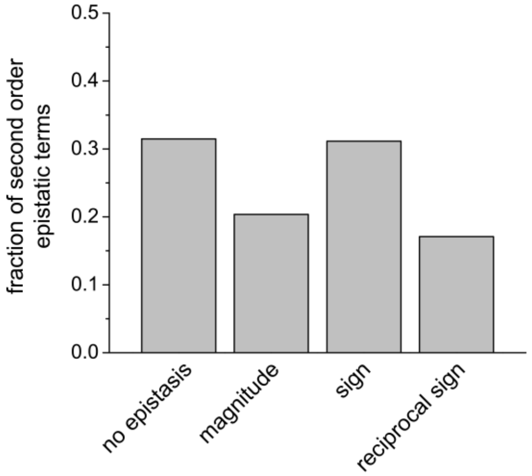

Supplementary Figure 11: Distribution of epistasis type amongst the pairwise background averaged epistatic terms. The type of epistatic motif (categories) between mutations at a pair of positions determines whether these mutations can be incorporated by an evolutionary process proceeding by single mutation steps[11,12]. Of the four categories, sign epistasis and reciprocal sign epistasis are the extreme forms that limit the accessible trajectories; their prevalence is a direct measure of ruggedness of the fitness landscape[13,14]. Shown here are the frequencies of each epistatic motif amongst all significant pairwise terms, indicating substantial extreme epistasis.

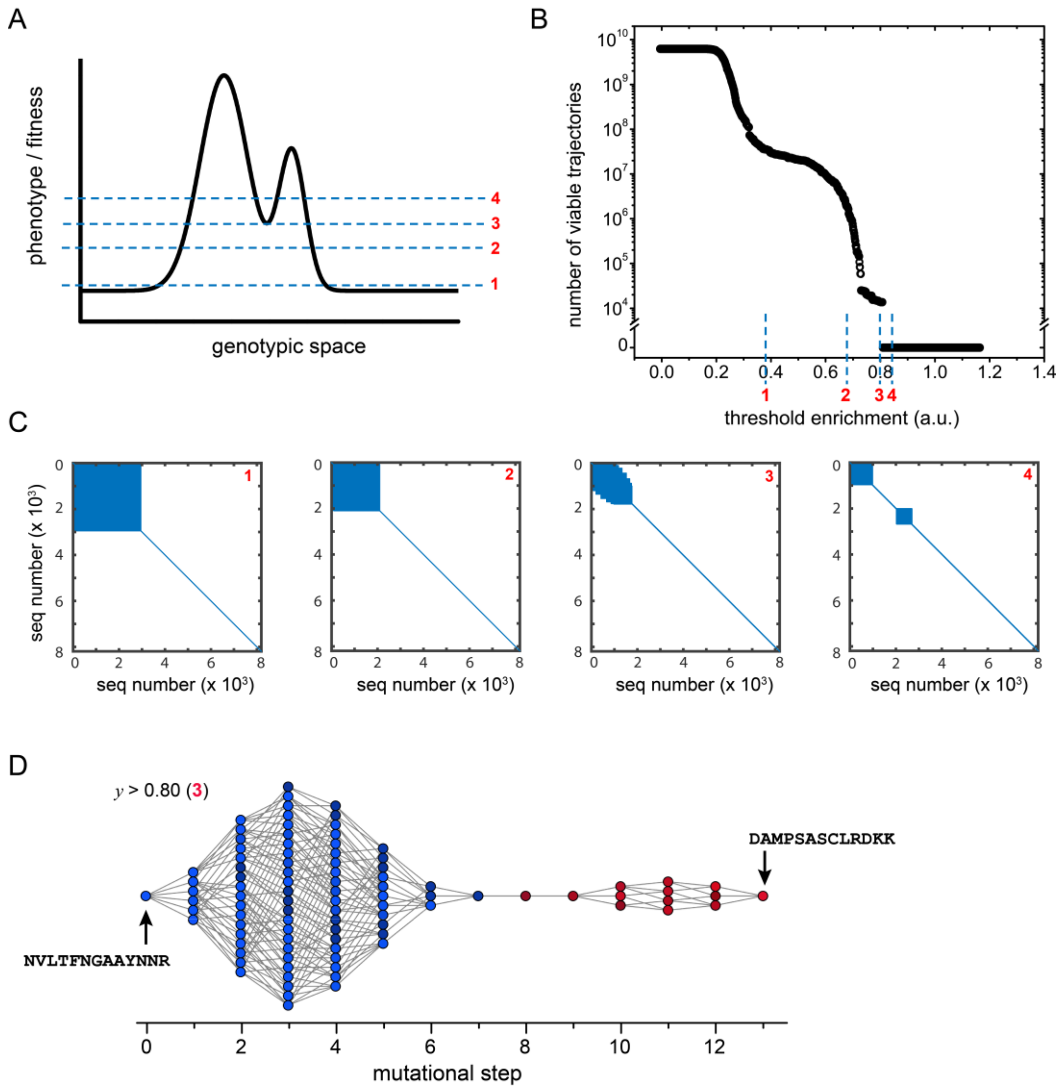

Supplementary Figure 12: Functional connectivity of the sequence space. (**A**), A schematic illustrating the concept of genotypic connectivity as a function of different phenotypic thresholds (marked in red, 1-4). The solution space is connected up to threshold 3. (**B**) The number of single-step trajectories as a function of threshold brightness for the data; thresholds corresponding to the cartoon in panel A are indicated. Note that the solution space becomes disconnected (zero viable paths) at threshold 3. (**C**) Genotypic "connectograms", a graphical representation of the single step functional connectivity of the sequence space as a function of threshold (red numbers corresponding to panel B) (see Methods for computational process). The graphs show that threshold 3 represents the critical point after which the solution space breaks and is not fully connected. (**D**), The structure of the solution space at the threshold for functional connectivity. As in Figure 6, the space is dumbbell-shaped, but with the neck linking the space now defined by an ordered series of single mutants. These genotypes (steps 7-9) harbor a reciprocal sign epistatic motif, which defines the strict order in which these mutations must come in order to be functionally connected. Note that at this threshold (y=0.80), the starting and ending genotypes shown here are not the parental ones.

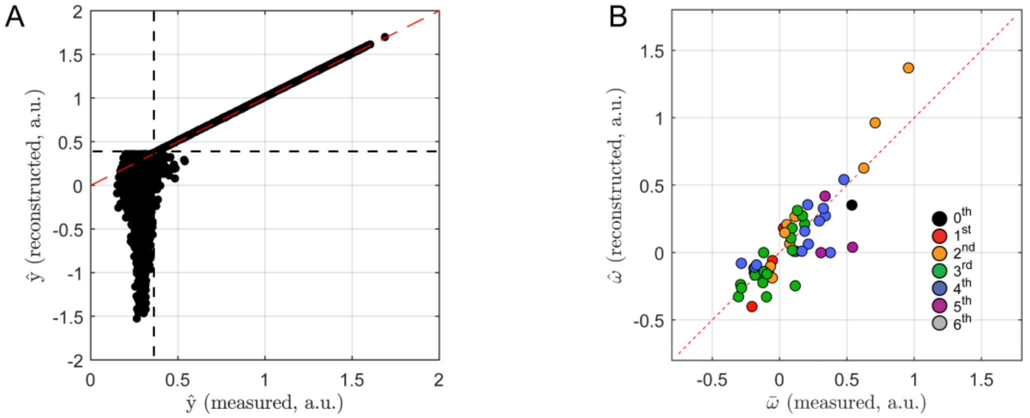

Supplementary Figure 13: Releasing epistatic constraints below the lower fluorescence detection limit. Every observable trait will exhibit overall nonlinearities. These can be either intrinsic, such as a saturating organismal fitness as a function of metabolic enzyme activity, or extrinsic, introduced by a limited observation range, or both. Depending on the definition of the quantity of interest, such nonlinearities can lead to an overestimate of the prevalence of epistasis. In this work we have minimized non-linearities using a linear-nonlinear optimization[4,5] (see main text and Methods). In this supplementary figure we specifically explore the effect of the lower detection limit in our FACS-seq approach, by performing an alternative analysis, assessing which epistatic terms are relevant for reproduction of the data in the linear regime only (Shen and Poelwijk, in preparation). This approach is based on reconstruction of the measured data, using a mildly sparsifying optimization, where data points below the limit of detection do not contribute to the loss term as long as their reconstructed values are also below the limit of detection. Mathematically, this is achieved by minimizing

$$\|\bar{\omega}\|_1 + \lambda \left\| \frac{(\boldsymbol{\Omega}^{-1}\bar{\omega} - \tau) + \mathrm{abs}(\boldsymbol{\Omega}^{-1}\bar{\omega} - \tau)}{2} - \frac{(\bar{y} - \tau) + \mathrm{abs}(\bar{y} - \tau)}{2} \right\|_2^2,$$

where $\bar{y}$, $\bar{\omega}$, $\boldsymbol{\Omega}$, are respectively the data vector, epistasis vector, and epistasis operator, as defined throughout this work, and τ is the threshold value below which data will not contribute to the loss term. λ is the regularization parameter, here with a large value, to favor the L2 term. This optimization is insensitive to the numeric values of data under the detection limit, but does require that those points are reconstructed in that regime. (**A**) Data reconstructed using the above method, $\hat{y}$, versus the brightness values $\bar{y}$ as given in Fig. 1C. As intended, we observe a near perfect fit (red dashed line) in the linear range above the detection threshold τ (black dashed lines, see also Supplementary Fig. 2), and an unconstrained distribution of data in $\hat{y}$ below the threshold. (**B**) Comparison of the values of the most prominent epistatic contributions as defined in Fig. 3B with the epistatic terms obtained in the above approach, colored by epistatic order of the terms. Good correlation between the two approaches indicates that in our analysis of the brightness data the lower detection limit does not play a dominant role.

References:

1 Poelwijk, F. J., de Vos, M. G. & Tans, S. J. Tradeoffs and optimality in the evolution of gene regulation. *Cell* **146**, 462-470, (2011).

2 de Boer, H. A., Comstock, L. J. & Vasser, M. The tac promoter: a functional hybrid derived from the trp and lac promoters. *Proc Natl Acad Sci U S A* **80**, 21-25, (1983).

3 Kinney, J. B., Murugan, A., Callan, C. G., Jr. & Cox, E. C. Using deep sequencing to characterize the biophysical mechanism of a transcriptional regulatory sequence. *Proc Natl Acad Sci U S A* **107**, 9158-9163, (2010).

4 Otwinowski, J. & Nemenman, I. Genotype to phenotype mapping and the fitness landscape of the E. coli lac promoter. *PLoS One* **8**, e61570, (2013).

5 Poelwijk, F. J. Context-Dependent Mutation Effects in Proteins. *Methods Mol Biol* **1851**, 123-134, (2019).

6 Poelwijk, F. J., Krishna, V. & Ranganathan, R. The Context-Dependence of Mutations: A Linkage of Formalisms. *PLoS Comput Biol* **12**, e1004771, (2016).

7 Stadler, P. in *Biological Evolution and Statistical Physics* 187-207 (Springer-Verlag, 2002).

8 Weinberger, E. D. Fourier and Taylor-Series on Fitness Landscapes. *Biol Cybern* **65**, 321-330, (1991).

9 Weinreich, D. M., Lan, Y., Wylie, C. S. & Heckendorn, R. B. Should evolutionary geneticists worry about higher-order epistasis? *Curr Opin Genet Dev* **23**, 700-707, (2013).

10 Candès, E. & Wakin, M. An Introduction to Compressive Sensing. *Signal Processing Magazine, IEEE* **25**, 21-30, (2008).

11 Weinreich, D. M., Watson, R. A. & Chao, L. Perspective: Sign epistasis and genetic constraint on evolutionary trajectories. *Evolution* **59**, 1165-1174, (2005).

12 Poelwijk, F. J., Kiviet, D. J., Weinreich, D. M. & Tans, S. J. Empirical fitness landscapes reveal accessible evolutionary paths. *Nature* **445**, 383-386, (2007).

13 Szendro, I. G., Schenk, M. F., Franke, J., Krug, J. & De Visser, J. A. G. M. Quantitative analyses of empirical fitness landscapes. *Journal of Statistical Mechanics: Theory and Experiment* **1**, P01005, (2013).

14 Poelwijk, F. J., Tanase-Nicola, S., Kiviet, D. J. & Tans, S. J. Reciprocal sign epistasis is a necessary condition for multi-peaked fitness landscapes. *J Theor Biol* **272**, 141-144, (2011).
