# Supplementary material for: Learning the pattern of epistasis linking genotype and phenotype in a protein
Source: Nat Commun. 2019 Sep 16;10:4213. doi: 10.1038/s41467-019-12130-8 (PMC6746860; doi:10.1038/s41467-019-12130-8)
Supplement: Supplementary file 3 — Description of Additional Supplementary Files [file 41467_2019_12130_MOESM3_ESM.pdf]

Description of Additional Supplementary Files

Supplementary Software 1: Computational scripts for sequence data processing and data analysis. Description of the main scripts is as follows:

a) Raw data processing scripts:

- GetSeqandQual.bat: Linux script extracting sequences and quality scores according to the expected sequence mask.
- scriptQualUnique.m: MATLAB script reading the extracted sequences and quality scores and filtering the uniqueness bar based on a Qscore cutoff.
- ExtrBars.bat: Linux script keeping/deleting sequences based on the pass/fail flag from the Qscore filtering in the previous step, and extracting the bar codes.
- scriptBars2Brightness.m: MATLAB script processing the extracted bar codes, performing uniqueness bar error correction, normalization, linear-nonlinear scaling, and adding pseudocounts to zero brightness phenotypes. Outputs the brightness variables.

b) Analysis scripts:

- scriptFPAnalysisZ.m: MATLAB script performing all analysis described in this work and reproducing the main figures.

Supplementary Data 1: Construction strategy; primers and construction fragments.
Supplementary Data 2: Constructed DNA plasmid sequence.
Supplementary Data 3: Brightness data.
Supplementary Data 4: Brightness data replicate.
Supplementary Data 5: Sanger sequenced barcodes and mutable positions.
